# Prognostic Outcomes and Predictive Factors in Non-Metastatic Castration-Resistant Prostate Cancer Patients Not Treated with Second-Generation Antiandrogens

**DOI:** 10.3390/biomedicines12102275

**Published:** 2024-10-08

**Authors:** Yu-Jen Wang, Chi-Shin Tseng, Chao-Yuan Huang, Chung-Hsin Chen, So-Meng Wang, Kuo-How Huang, Po-Ming Chow, Yeong-Shiau Pu, Jeff Shih-Chieh Chueh, Shiu-Dong Chung, Jason Chia-Hsien Cheng

**Affiliations:** 1 Department of Radiation Oncology, Fu Jen Catholic University Hospital, New Taipei City 24352, Taiwan; 2 School of Medicine, College of Medicine, Fu Jen Catholic University, New Taipei City 242062, Taiwan; 3 Departments of Urology, National Taiwan University College of Medicine and Hospital, Taipei 100012, Taiwan; 4 Division of Urology, Department of Surgery, Far Eastern Memorial Hospital, New Taipei City 220216, Taiwan; 5 Department of Nursing, College of Healthcare & Management, Asia Eastern University of Science and Technology, Taipei 220303, Taiwan; 6 Division of Radiation Oncology, Department of Oncology, National Taiwan University College of Medicine and Hospital, Taipei 100012, Taiwan; 7 Graduate Institutes of Oncology, National Taiwan University College of Medicine, Taipei 100012, Taiwan; 8 Graduate Institutes of Clinical Medicine, National Taiwan University College of Medicine, Taipei 100012, Taiwan

**Keywords:** prostate cancer, prostate-specific antigen, non-metastatic castration-resistant prostate cancer, metastasis, second-generation antiandrogens

## Abstract

**Background/Objectives:** Patients with non-metastatic castration-resistant prostate cancer (nmCRPC) and high-risk features frequently have progression to life-threatening metastasis without second-generation antiandrogens. This study investigated nmCRPC patients for the survival and prognostic factors from a cohort before the approved use of second-generation antiandrogens. **Methods:** From March 2016 to January 2021, 326 patients treated with second-generation antiandrogens for metastatic castration-resistant prostate cancer (mCRPC) or metastatic castration-sensitive prostate cancer were retrieved. Forty-four patients experiencing nmCRPC with no use of second-generation antiandrogens were reviewed. The prognostic factors, at initial diagnosis or at nmCRPC, associated with metastasis-free survival (MFS) and overall survival (OS) were analyzed. **Results:** The median follow-up time after nmCRPC was 46 months. The median PSA level at nmCRPC was 2.7 ng/mL. Thirty-eight of forty-four patients with nmCRPC had a PSA doubling time (PSADT) of 10 months or shorter, and the median PSADT was 4 months. The median OS from nmCRPC was 53 months, and the median interval for nmCRPC patients progressing to mCRPC was 20 months. Upon univariate analysis, PSADT < 10 months (*p* = 0.049) and the very-high-risk group at the initial diagnosis (*p* = 0.043) were associated with significantly shorter post-nmCRPC MFS. The very-high-risk group (*p* = 0.031) was associated with significantly worse post-nmCRPC OS. In terms of survivals from the initial diagnosis of prostate cancer, Gleason grade ≥ 8 was the only independent factor with MFS and OS. **Conclusions:** Without second-generation antiandrogens, nmCRPC patients with PSADT <10 months and in the initial very-high-risk group developed subsequent mCRPC in a significantly faster fashion. Patients of the very-high-risk group had shorter survival rates after nmCRPC.

## 1. Background/Objectives

Non-metastatic castration-resistant prostate cancer (nmCRPC) is defined as a rising prostate-specific antigen (PSA) concentration, despite the castration levels of testosterone with ongoing androgen-deprivation therapy (ADT) or orchiectomy, and no detectable metastasis by conventional imaging modalities [1]. Patients with nmCRPC have a disease progression to metastasis and have the risk of developing metastasis-related symptoms and morbidity, eventually dying of their disease. Although patients with nmCRPC are generally asymptomatic from their disease, they are often of an old age and require long-term concomitant medication for chronic comorbidities. Therefore, early intervention and effective treatment are mandated, especially for nmCRPC patients with a higher risk of disease progression and worse prognosis of survival [2].

Three second-generation antiandrogens or next-generation androgen receptor inhibitors have been approved for the treatment of nmCRPC on the basis of phase 3 trials, including the Androgen Receptor Antagonizing Agent for Metastasis-free Survival (ARAMIS) trial for darolutamide [3], the Selective Prostate Androgen Receptor Targeting with ARN-509 (SPARTAN) [4] trial for apalutamide [5], and the Safety and Efficacy Study of Enzalutamide in Patients With Non-Metastatic Castration-Resistant Prostate Cancer (PROSPER) trial for enzalutamide [6,7].

It is generally accepted that a poor prognosis of patients with nmCRPC is most frequently suggested by a shorter PSA doubling time (PSADT). After the emergence of metastasis, the outlook for survival outcomes decreases [8]. However, the factors contributing to nmCRPC in patients with locally advanced prostate cancer and the prognostic factors related to nmCRPC have not been thoroughly studied from limited available clinical data outside randomized controlled trials. Identifying clinical prognostic factors that influence the development from nmCRPC to metastatic castration-resistant prostate cancer (mCRPC) is crucial for improving patient outcomes. We retrospectively collected data on patients with mCRPC who experienced the clinical nmCRPC course and analyzed their clinical outcomes and prognostic characteristics. We hope to find out the patient populations that are prone to develop nmCRPC and the factors that affect survival after nmCRPC.

## 2. Methods

### 2.1. Patients

We retrospectively reviewed the medical records of all patients who received the second-generation antiandrogens under the indication of mCRPC or metastatic castration-sensitive prostate cancer (mCSPC) at our institution between March 2016 and January 2021. The patients who received second-generation antiandrogens met the following conditions as follows: patients with mCRPC who were symptom-free or had mild symptoms after failing ADT and did not received chemotherapy, or those with drug- or surgically resistant mCRPC who underwent at least two unsuccessful treatments with docetaxel. The inclusion criteria of this study were nmCRPC patients with non-metastatic disease at the initial diagnosis of prostate cancer and had a clinical course of nmCRPC before mCRPC. The National Comprehensive Cancer Network (NCCN) offers guidelines for risk classification at the initial diagnosis [9]. The criteria of nmCRPC was a baseline PSA level of at least 2 ng/mL and testosterone level lower than 0.5 ng/mL and no imaging evidence of distant metastasis outside the pelvis. Patients with nmCRPC and PSADT of 10 months or less were classified as the high-risk group. All patients received a radionuclide bone scan of the whole body and computed tomography (CT) or magnetic resonance imaging (MRI) of the abdomen and pelvis or a prostate-specific membrane antigen position emission tomography (PSMA-PET) scan to confirm a non-metastatic status for the diagnosis of nmCRPC. The study was approved by our Institutional Review Board. A flow chart for screening patients for this study is shown in Supplementary Figure S1.

### 2.2. Treatments

Androgen deprivation therapy (ADT) was administered in all patients experiencing nmCRPC in this study. During the study period, the choice of second-generation antiandrogens for treatment, based on the professional judgment of the physicians, was approved only in patients with mCRPC or mCSPC.

### 2.3. Follow-Up

Patients were followed up every 3 months with serum PSA and testosterone, a CT or MRI of the abdomen and pelvis, and a whole body bone scan. Follow-up duration, survival time, and event time were calculated from the time of the original diagnosis of nmCRPC. The total survival ourcome was defined as the period from the initial diagnosis of prostate cancer to the date of death or last follow-up visit. The post-nmCRPC survival outcome was defined as the period from the diagnosis of nmCRPC to death or last follow-up visit. The post-nmCRPC metastasis-free survival (MFS) outcome was defined as the period between nmCRPC and mCRPC. Kaplan–Meier analysis was performed to calculate survival rates. 

### 2.4. Statistics

A descriptive analysis was performed by computing ranges, averages, median values, and standard deviations. A comparison of continuous variables was carried out using a two-tailed unpaired Student’s *t*-test. For analyzing contingency tables, both Chi-square and Fisher’s exact tests were utilized. The log-rank test was applied to identify the prognostic factors that impacted survival. In the multivariate analysis, all prognostic variables that were found to be either significant or borderline significant through univariate analysis were included using the Cox proportional hazard regression model. Statistical significance was determined as *p* < 0.05. All statistics were calculated with IBM SPSS Statistics for Windows, version 24 (IBM Corp., Armonk, NY, USA) 

## 3. Results

### 3.1. Patient Characteristics

In total, 83 patients were identified from 319 patients taking the second-generation antiandrogens with no metastasis at the time of biochemical failure. Two hundred and twenty mCSPC patients at the initial diSagnosis and sixteen mCSPC patients at biochemical failure were excluded. Among the 83 patients, 44 patients met the criteria of nmCRPC at biochemical failure and formed the basis of this study after excluding 39 patients with upfront mCRPC. The median age at the time of the initial diagnosis was 69 years (range: 45–87), and for nmCRPC, it was 77 years (range: 57–91). Thirty-two patients had initial clinical T3 disease (72.7%) and 6 had T4 disease (13.6%). Six patients (13.6%) had a Group 4 Gleason score and twenty (45.4%) had a score of 9 or 10. All patients received ADT and 26 patients had undergone local RT. The median time from primary treatment to nmCRPC was 108 months (range: 45–87). The median PSA level when meeting the criteria of nmCRPC was 2.7 ng/mL (range: 2.0–9.8). With a progression from nmCRPC to mCRPC, the median PSA level at subsequent mCRPC was 14 ng/mL (range: 2.7–46 ng/mL). The median PSADT for nmCRPC was 4 months (range: 1–17). Based on PSADT, 39 patients were classified as high risk (≤10 months) and 6 patients as low risk (>10 months). The baseline characteristics are shown in Table 1.

### 3.2. Survival Outcomes and Patterns of Failure

With a median follow-up period of 46 months (range: 14–98), the 3-year post-nmCRPC overall survival rate of 44 patients diagnosed with nmCRPC was 78% (Supplementary Figure S2). The median overall survival from nmCRPC was 53 months (range: 4–99). The median survival from the initial diagnosis of prostate cancer was 129 months (range: 40–295). The median time for the nmCRPC patients progressing to mCRPC was 20 months (range: 3–73). At the time of mCRPC, 21 patients had bone metastasis, 14 had distant lymph node metastasis, 4 had both distant nodal metastasis and bone metastasis, and 5 patients had distant metastasis at other sites including the lung, liver, and bladder. 

### 3.3. Prognostic Factors on Metastasis-Free Survival

Upon completing univariate analysis, PSADT <10 months (*p* = 0.049) and the very-high-risk group at the initial diagnosis (*p* = 0.043, Supplementary Figure S3) were associated with significantly shorter post-nmCRPC MFS. In contrast, a Gleason score ≥8 (*p* = 0.003) and no previous local treatment (*p* = 0.023) were associated with significantly shorter total MFS from the initial diagnosis (Table 2). Upon completing multivariate analysis, none of these factors reached statistical significance for post-nmCRPC MFS, but the Gleason score remained significant (HR = 2.70, *p* = 0.011) for total MFS from the initial diagnosis of prostate cancer (Table 3).

### 3.4. Prognostic Factors on Overall Survival

**Figure 1:**
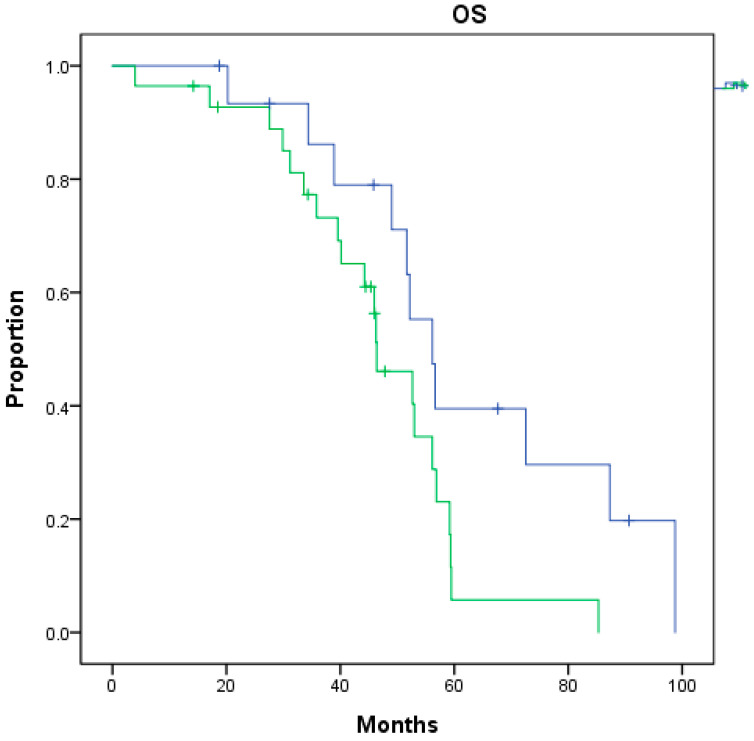
Overall survival (OS) from nmCRPC between nmCRPC patients of very-high-risk group (green) or not (blue) at initial diagnosis (*p* = 0.031).

Upon completing univariate analysis, the very-high-risk group at the initial diagnosis (*p* = 0.031) was associated with significantly worse post-nmCRPC survival (Figure 1). In contrast, a Gleason score ≥ 8 (*p* = 0.038) was associated with significantly worse total overall survival from the initial diagnosis (Table 2). Upon completing multivariate analysis, none of these factors reached statistical significance for either post-nmCRPC survival or total overall survival from the initial diagnosis (Table 3).

## 4. Discussion

This retrospective analysis investigated the clinical factors that influence the prognosis of nmCRPC not treated with second-generation antiandrogens and its progression to mCRPC. The findings revealed that patients diagnosed with nmCRPC were at a high or very high risk of prostate cancer diagnosis, and their progression to mCRPC was faster when the PSADT was less than or equal to 10 months at the nmCRPC diagnosis or, at their initial diagnosis, they were was placed into the very-high-risk group. Furthermore, patients who were initially diagnosed with prostate cancer in the very-high-risk group or had a PSADT ≤ 10 months had worse overall survival. These results imply that patients with very-high-risk prostate cancer at the initial diagnosis are at a greater risk of developing nmCRPC and have a poorer prognosis if they progress to nmCRPC.

Prior to the era of randomized studies investigating the use of second-generation antiandrogens for nmCRPC, there were only limited prognostic data to provide the frequently ignored information about this group of patients. Especially with no effective treatment options for these patients, this clinical issue has been often overlooked. One of the strengths of our study is that we have been able to provide complementary data for this group of patients. The detailed prognosis of nmCRPC has not been thoroughly investigated, partly due to the lack of effective treatment in the past [10]. Fortunately, we now have the medications proven effective to treat these nmCRPC patients. Furthermore, if progression to the next stage of mCRPC can be prevented or postponed, such clinical importance should be emphasized.

The patient group in this study was close to the placebo group in the ARAMIS, SPARTAN, and PROSPER trials, which evaluated nmCRPC patients treated with second-generation antiandrogens [11]. The median age at diagnosis, as well as the proportion of patients with high-risk features such as a short PSADT and high Gleason score, is consistent with patients reported in these trials. However, it is of note that the patient population in this study was relatively small compared to those in the three large trials. The proportion of patients with initial clinical T3 disease in this study was higher than in the SPARTAN trial (36.7% vs. 72.7%) and our patients were also older (77 y/o) than those in these trials. In terms of survival outcomes, the overall survival rate at three years for nmCRPC patients in this study was 78%, which was similar to the rates reported in the ARAMIS and SPARTAN trials at the same time point. The median interval of progression from nmCRPC to mCRPC was similar to the ARAMIS and SPARTAN trials. The significant prognostic factors identified in this study were consistent with those in the ARAMIS and SPARTAN trials, including PSADT and initial disease stage. A simple comparison group of 40 patients, with a median follow-up of 31 months, was treated with second-generation hormone drugs at our institute. Their 2-year overall survival and metastasis-free survival rates were 100% and 88%, respectively (Supplementary Figure S4).

Our study also compared low-risk nmCRPC patients (with a PSADT longer than 10 months) and found the interval of progression from nmCRPC to mCRPC comparable with another large retrospective study [12]. Notably, in the analysis of ARAMIS, the PSADT was able to differentiate prognosis after the progression to mCRPC [13]. However, our study showed no significant difference in post-mCRPC overall survival. This means that it is especially important to treat and stop the disease at nmCRPC and prevent its progression to mCRPC.

It is interesting to note that if patients were initially at a very high risk at the initial diagnosis, they experienced a significantly shorter interval from nmCRPC to mCRPC and worse overall survival. Patients who belonged to the very-high-risk group have particularly poor oncological outcomes [14], and therefore, aggressive managements should be initiated earlier [15]. Besides frequently reported PSADTs <10 months, our study demonstrated evidence of an additional prognostic factor for the very-high-risk group at the initial diagnosis and provided physicians with useful information regarding patient follow-up for the management of nmCRPC.

The study is limited by its retrospective design and its relatively small sample size, which may limit the generalizability of the findings. The control groups of the three clinical trials had a total of 1428 patients, and we could expand the population size of our study by showing the overall survival rates of around 56 months to 60 months and metastatic survival of around 14.7 months to 18.4 months, respectively, in the absence of second-generation antiandrogens (Supplementary Table S1) [3,5,6,16]. The statistical significance might be underestimated with few patients and events. In addition, to confirm the metastasis, most patients still used conventional imaging methods, which may not be able to as accurately diagnose metastasis as PSMA-PET [17]. Based on a previously published study, PSMA-PET was up to 98% positive with metastasis in high-risk nmCRPC patients [18]. However, it has not yet been included in the current guidelines or in the three randomized trials as part of the routine examinations for nmCRPC [19]. This study highlights the importance of identifying patients with very-high-risk prostate cancer at the initial diagnosis and closely monitoring their PSADT to detect the development of nmCRPC as early as possible. This could lead to timely intervention and potentially improve outcomes. Further large-scale research is needed to confirm these findings and investigate the potential interventions to prevent or delay the progression of nmCRPC to mCRPC.

## 5. Conclusions

Patients initially diagnosed with very-high-risk prostate cancer and with a PSADT ≤ 10 months for nmCRPC have a significantly shorter interval of the progression to mCRPC. Patients with very-high-risk prostate cancer have a worse overall survival rate after nmCRPC.

## Figures and Tables

**Table 1:** Clinical characteristics of 44 patients with non-metastatic castration-resistant prostate cancer (nmCRPC).

Variable		Patient Number	Percent
Total		44	100
Age at diagnosis			
	<65	16	36.4
	65–75	15	34.1
	>75	13	29.5
Age at nmCRPC			
	<65	5	11.4
	65–75	13	29.5
	>75	26	59.1
Initial clinical T stage			
	T2	6	13.6
	T3	32	72.7
	T4	6	13.6
Initial risk group			
	Intermediate risk	7	15.9
	High risk	10	22.7
	Very high risk	27	61.4
Images to confirm non-metastatic status			
	Bone scan	20	45.4
	CT	14	31.8
	MRI	7	15.9
	PSMA-PET	8	18.1
Previous local treatment			
	Radical prostatectomy	6	13.6
	Radiotherapy	26	59.1
Initial PSA level			
	<10 ng/mL	10	22.7
	10–20 ng/mL	7	15.9
	>20 ng/mL	27	61.5
Sites of distant metastasis at mCRPC			
	Bone metastasis	21	47.7
	Distant lymph nodes	14	31.8
	Distant lymph nodes and Bone metastasis	4	9.1

PSMA-PET: prostate-specific membrane antigen positron emission tomography.

**Table 2:** Univariate analyses of the prognostic factors on metastasis-free survival (MFS) post-non-metastatic castration-resistant prostate cancer (nmCRPC), total MFS from initial diagnosis, post-nmCRPC overall survival (OS), and total OS from the initial diagnosis of prostate cancer.

Variable	Post-nmCRPC MFS (Month)	*p* Value	Total MFS (Month)	*p* Value	Post-nmCRPC OS (Month)	*p* Value	Total OS (Month)	*p* Value
PSA doubling time (months)								
≥10 months	36.0	0.049	135.6	0.368	67.6	0.132	185.8	0.210
<10 months	20.3		79.2		49.0		114.3	
Initial risk group at initial diagnosis								
Very-high-risk group	19.5	0.043	75.1	0.207	48.4	0.031	104.5	0.141
High-risk group	36.0		86.0		56.2		148.5	
Initial T stage								
T3 or T4	20.8	0.56	84.1	0.653	52.5	0.415	114.3	0.759
T2	19.5		123.6				133.2	
Initial Gleason Score								
≥8	19.5	0.165	74.6	0.003	46.4	0.447	100.5	0.038
<8	36.1		129.0		52.7		185.8	
Initial PSA level								
≥20	20.3	0.882	72.9	0.084	52.2	0.429	114.4	0.092
<20	26.4		111.1		56.2		149.7	
Initial treatment								
RP or RT	19.5	0.424	135.1	0.023	52.7	0.725	185.8	0.074
No RP or RT	36.1		74.6		52.2		104.5	
PSA level at nmCRPC								
≥3	18.8	0.850	17.6	0.602	46.4	0.366	97.0	0.231
<3	20.8		15.4		52.7		148.5	

nmCRPC: non-metastatic castration-resistant prostate cancer. RP: radical prostatectomy.

**Table 3:** Multivariate analyses of the prognostic factors on metastasis-free survival (MFS) post-non-metastatic castration-resistant prostate cancer (nmCRPC), MFS from initial diagnosis, post-nmCRPC overall survival (OS), and total OS from the initial diagnosis of prostate cancer.

Variable	Post-nmCRPC MFS HR	*p* Value	Total MFS HR	*p* Value	Post-nmCRPC OS HR	*p* Value	Total OS HR	*p* Value
PSA doubling time (months)								
≥10 months	0.69–6.10	0.196			0.6	0.272		
<10 months					−7.4			
Initial risk group at initial diagnosis								
Very-high-risk group	0.75–3.32	0.227			0.8–4.7	0.142	0.28–1.60	0.37
Initial Gleason Score			1.25–5.8	0.011				
≥8							0.65–4.57	0.27
<8								
Initial treatment			0.26–1.05	0.067				
RP or RT							0.77–4.10	0.18
No RP or RT								
Initial PSA level >20			1–3.69	0.050				

nmCRPC: non-metastatic castration-resistant prostate cancer. RP: radical prostatectomy.

## Data Availability

The data that support the findings of this study are available on request from the corresponding author.

## Supplementary Materials

The following supporting information can be downloaded at https://www.mdpi.com/article/10.3390/biomedicines12102275/s1, Figure S1: Flow chart of nonmetastatic castration-resistant prostate cancer (nmCRPC) patients screened from patients taking the second-generation antiandrogens for metastatic castration-resistant prostate cancer (mCRPC). Figure S2: (A) Overall survival (OS) and (B) metastasis-free survival ((MFS) of 44 patients after non-metastatic castration-resistant prostate cancer (nmCRPC). Figure S3: Metastasis-free survival from nmCRPC between (A) patients with PSA doubling time ≤10 months (blue) and >10 months (green) (*p* = 0.049), and (B) patients of very high-risk group (blue) or not (green) at initial diagnosis of prostate cancer (*p* = 0.043); Figure S4: Metastasis-free survival ((MFS) of 40 patients after non-metastatic castration-resistant prostate cancer (nmCRPC) who treated with the second-Generation Antiandrogens. Table S1: Comparison of this study with three clinical trials (ARAMIS, SPARTAN, and PROSPER) on patient overall survival (OS) and metastatic free survival (MFS) from patients without taking the second-generation antiandrogens for non-metastatic castration-resistant prostate cancer (nmCRPC).
