# Supplementary material for: Prognostic Outcomes and Predictive Factors in Non-Metastatic Castration-Resistant Prostate Cancer Patients Not Treated with Second-Generation Antiandrogens
Source: Biomedicines. 2024 Oct 8;12(10):2275. doi: 10.3390/biomedicines12102275 (PMC11504664; doi:10.3390/biomedicines12102275)
Supplement: Supplementary file 1 [file biomedicines-12-02275-s001.zip › biomedicines-3205398-supplementary.pdf]

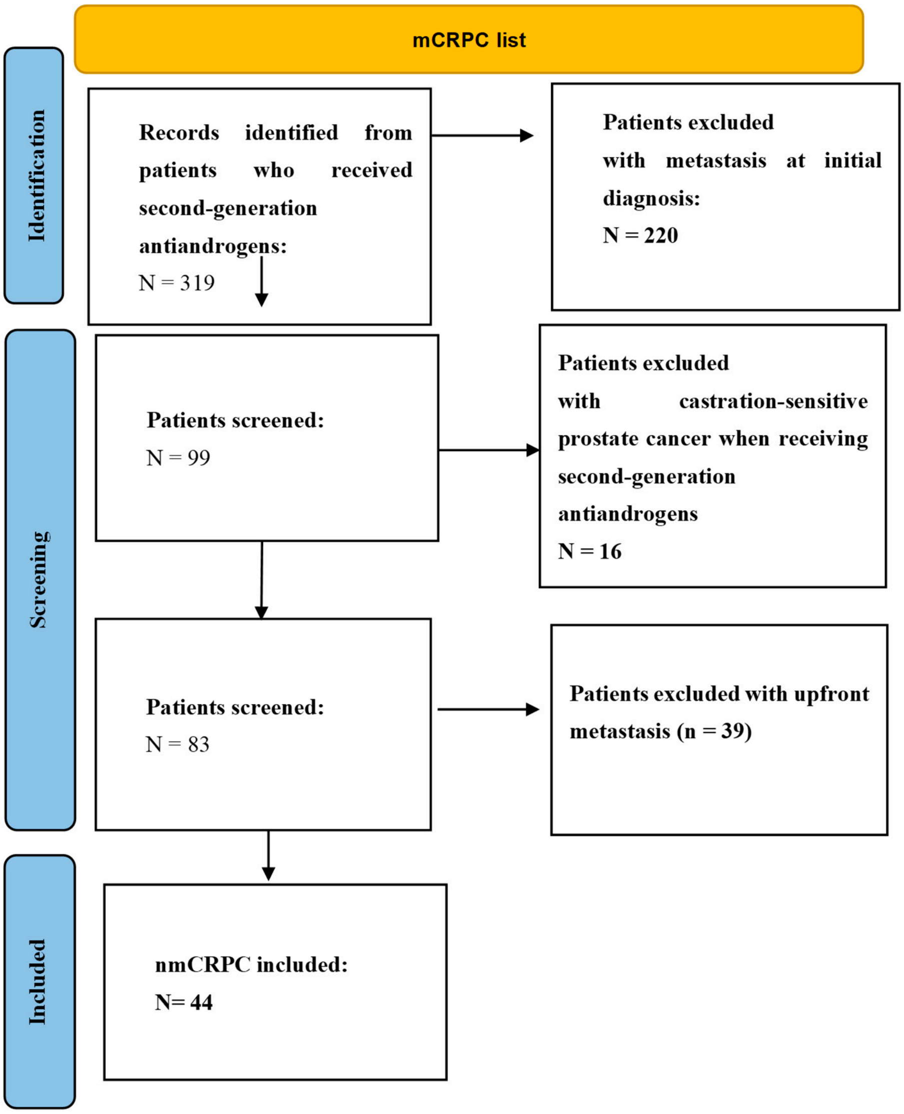

Figure S1. Flow chart of nonmetastatic castration-resistant prostate cancer (nmCRPC) patients screened from patients taking the second-generation antiandrogens for metastatic castration-resistant prostate cancer (mCRPC).

Table S1. Comparison of this study with the control groups of three clinical trials (ARAMIS, SPARTAN, and PROSPER) on patient overall survival (OS) and metastatic-free survival (MFS) from patients without the second-generation antiandrogens for non-metastatic castration-resistant prostate cancer (nmCRPC).

	Our study	ARAMIS	SPARTAN	PROSPER
Patient numbers	44	554	401	468
OS (months)	53		59.9	56.3
MFS (months)	20	18.4	16.2	14.7

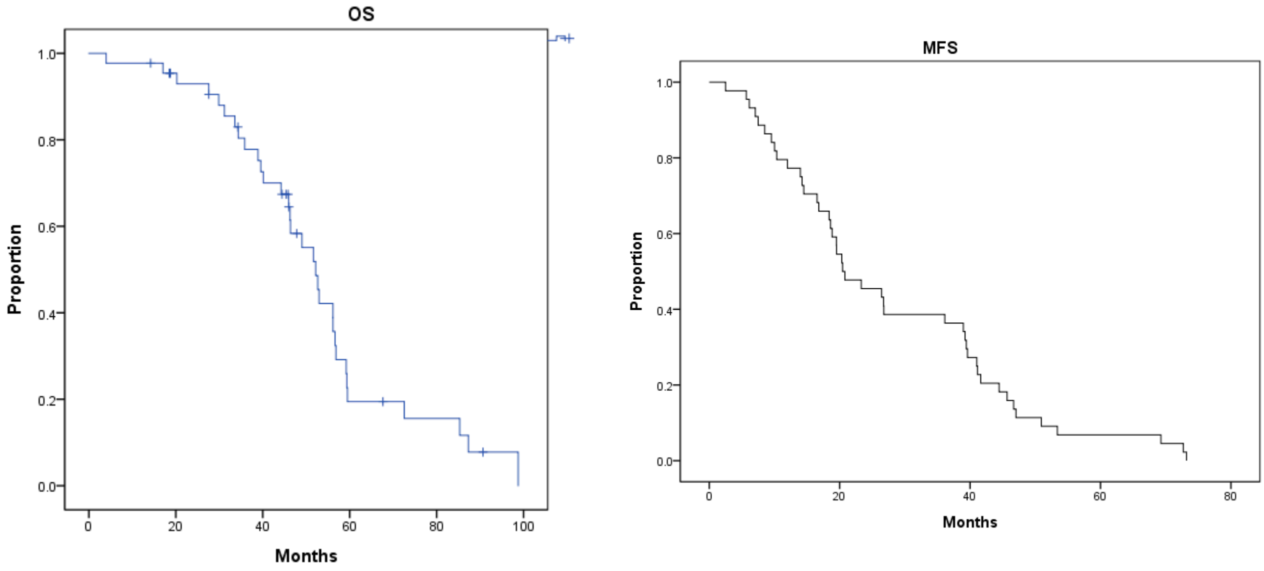

(A) **(B)**

Figure S2. (A) Overall survival (OS) and (B) metastasis-free survival ((MFS) of 44 patients after non-metastatic castration-resistant prostate cancer (nmCRPC).

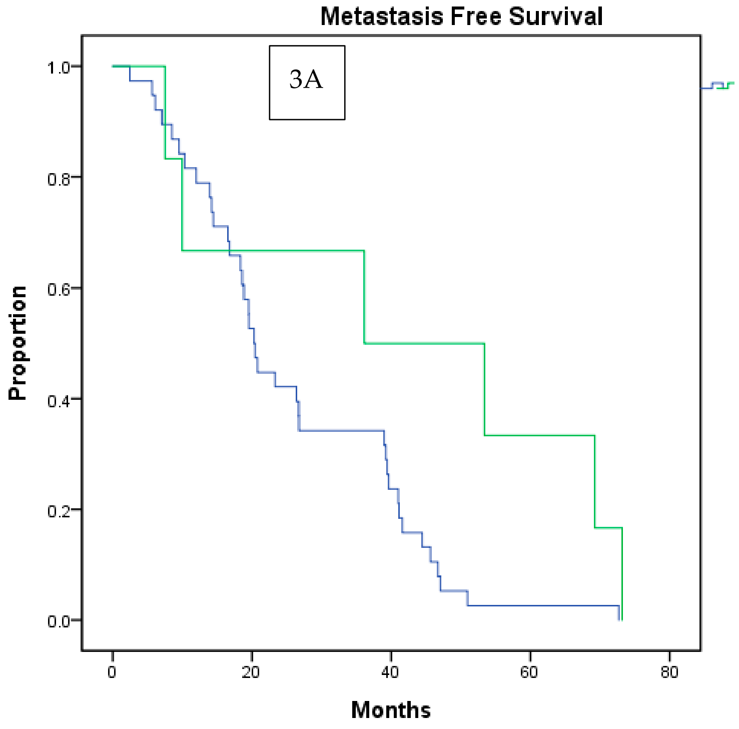

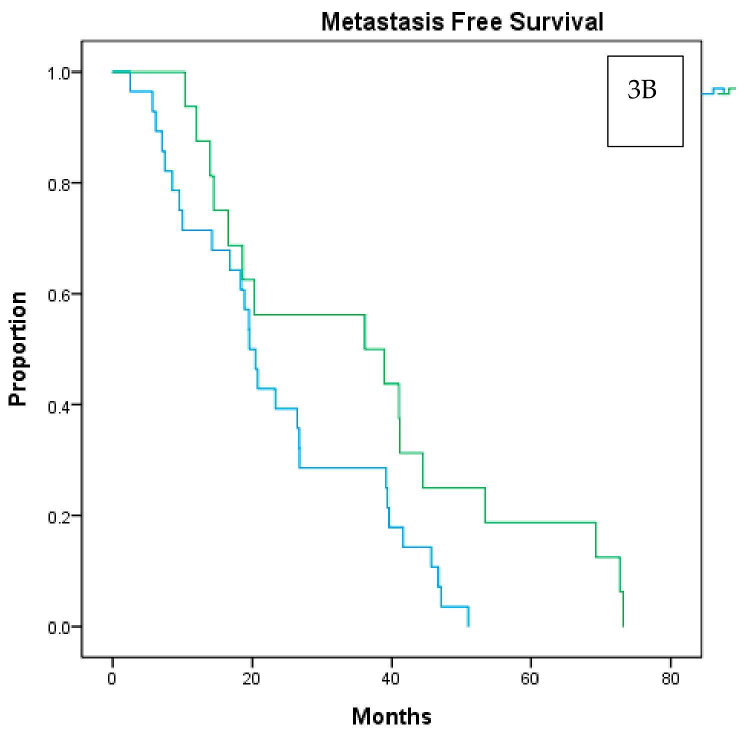

Figure S3. Metastasis-free survival from nmCRPC between (A) patients with PSA doubling time ≤10 months (blue) and >10 months (green) (P=0.049), and (B) patients of very high-risk group (blue) or not (green) at initial diagnosis of prostate cancer (P=0.043).

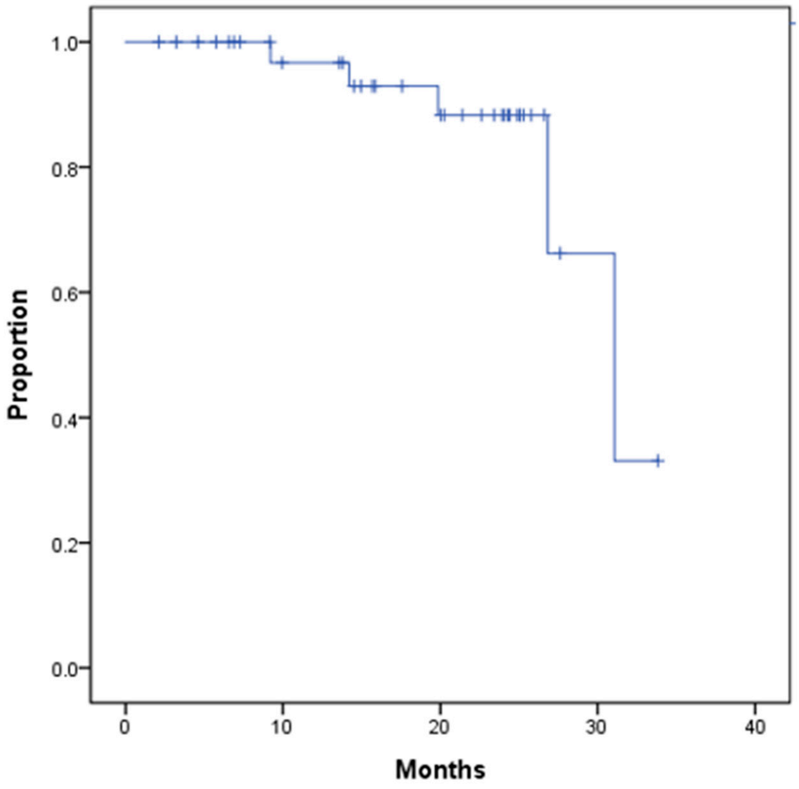

Figure S4. Metastasis-free survival ((MFS) of 40 patients after non-metastatic castration-resistant prostate cancer (nmCRPC) who were treated with the second-Generation Antiandrogens.
